# Visualisation tool for peptide fractionation data in proteomics: application to OFFGEL isoelectric focussing

**DOI:** 10.1186/1471-2105-11-371

**Published:** 2010-07-05

**Authors:** David-Olivier D Azulay, Hendrik Neubert, Mireia Fernández Ocaña

**Affiliations:** 1 Molecular Medicine, Pfizer Global Research and Development, Sandwich, UK; 2 Pharmacokinetics Dynamics and Metabolism, Pfizer Global Research and Development, Sandwich, UK; 3 Structural Biology and Biophysics, Pfizer Global Research and Development, Sandwich, UK

## Abstract

**Background:**

OFFGEL isoelectric focussing (IEF) has become a popular tool in proteomics to fractionate peptides or proteins. As a consequence there is a need for software solutions supporting data mining, interpretation and characterisation of experimental quality.

**Results:**

We can assess performance characteristics of OFFGEL IEF peptide fractionation in proteomics by generating plots of the overall fractionation patterns and the pairwise comparisons of adjacent fractions.

**Conclusions:**

A visualisation tool for peptide fractionation has been developed to support the evaluation of IEF data quality and can be implemented in proteomics research.

## Background

Most proteomics workflows from complex biological matrices require extensive sample processing at peptide or protein level to increase identification coverage. Recently, due to its separation capabilities, ease of use and relatively low cost, OFFGEL isoelectric focussing (IEF) has become a popular tool to fractionate proteins and peptides by their isoelectric point (pI) prior to LC-MS/MS [1,2]. The increase in the number of peptide identifications acquired from all fractions compared to the number derived from unfractionated samples demonstrates the value of this technology [3]. The IEF separation performance has been illustrated in detail for example by displaying in histograms the percentage of unique peptides identified in each fraction and the number of fractions in which each distinct peptide was found [4]. Furthermore, the correlation between estimated and experimental peptide pI has been demonstrated [5-7]. As an extension of these interpretation methods, this article presents a visualisation tool that illustrates the overall separation performance and displays the spread of peptides in common across adjacent IEF fractions. To evaluate this spread this tool enables sorting the peptide identifications by calculated pI, mass or MASCOT score. The visualisation tool also allows importing scores obtained from other search engines and can extract for each peptide different intrisic parameters, for example hydrophobicity [8] or a value describing the length of the pH range where the net charge of the peptide is below a selected threshold.

## Implementation

**Figure 1:**
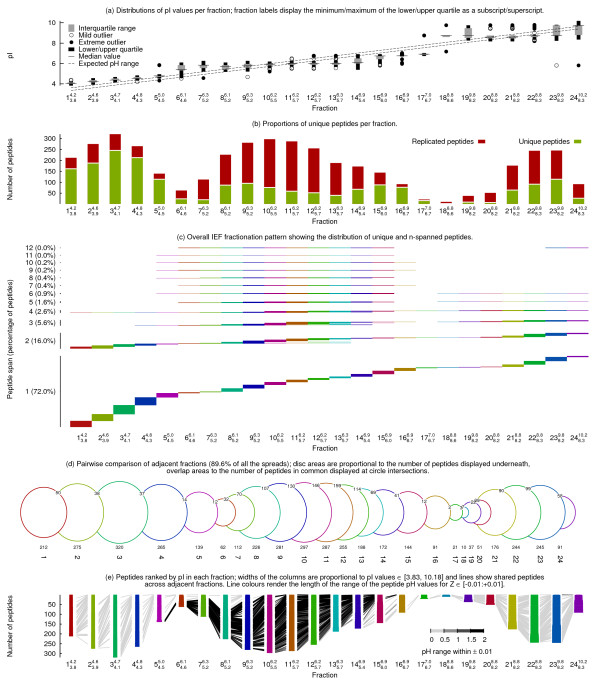
**Graphical output of the visualisation tool illustrating IEF separation performance**.

In order to fetch estimated peptide pIs and molecular weights from bioinformatics web sites like [9] or [10], a HTTP page retrieving package was mandatory and implemented in this tool. We opted for the Perl language and its HTTP request module [11,12]. The graphical rendering requires Gnuplot which is a plotting utility originally created to allow scientists to visualise mathematical functions and data [13]. All three pieces of software are freely available on a large number of platforms. The final output is a one page PNG (Portable Network Graphics), PDF (Portable Document Format) or Postcript file as illustrated in Figure 1.

The implementation relies on a Perl program whose output is a Gnuplot script that produces all the graphics. As inputs the tool reads a tab delimited text file containing the peptide sequences, estimated pIs, molecular weights and MASCOT scores and an optional tab delimited file containing the expected pH ranges for each fraction. A missing pI value or molecular weight automatically triggers the request of the corresponding information from the web site of choice.

The box plot (Figure 1a, [14]) and histogram (Figure 1b) graphics have been previously presented and generated with other programs [4,6,7]: these are included for completeness. In addition this program generates three new figures which have not been previously presented. Figure 1c showing the overall fractionation pattern is a bitmap picture. A short fixed length horizontal segment of pixels is filled if a peptide is present in a fraction such that its repetition in different fractions creates longer segments. The colours help count the number of fractions covered by long lines and are preserved in the other two figures. Since Figure 1d displays fractions as circles with variable diameters, the colours help align the fractions from the plots above and below. Intersecting points are computed and arcs of circles are drawn to render the overlap [15]. Finally Figure 1e is built from stacks of variable length horizontal segments correlated with estimated pI values: every peptide is displayed as a centered segment and is automatically joined by a line if detected in an adjacent fraction.

The net charge versus pH titration curve is calculated for each peptide in 0.1 pH unit increments across the pH range from 1 to 14 using the acid dissociation constants from [16]. Parameters such as the length of the pH range where the net charge is below a threshold value (*i.e*. ± 0.01) are then derived and displayed in Figure 1e with shades of grey for the connecting lines between columns.

## Results

The following data set was used: proteins from a Huh7 cell lysate were used as a model system; reduced, alkylated and digested with trypsin. Peptide separation was performed on a 3100 OFFGEL fractionator as described elsewhere [4]. A total of 24 fractions were collected and analysed on a nanoLC Ultimate 3000 (Dionex, UK) coupled on-line to a Qstar pulsar i mass spectrometer (Applied Biosystems, UK). Data were searched utilising MASCOT Version 2.1.04 (Matrix Science, London, UK) within the human taxonomy of the SwissProt R50 database. Identified peptide sequences were extracted together with mass, ion score and estimated pI (calculated with [9] here; where references and documentation is available).

A peptide detected in *n *distinct fractions is defined as *n*-spanned, one detected in a single fraction as unique. A box plot of peptide pI distributions per fraction (Figure 1a) shows that average pI values fit reasonably well with expected pH ranges [4,6,7,17]. Expected pH ranges for each fraction were obtained from the manufacturer and overlaid with experimental pI (dotted lines). A histogram that displays the percentage of unique peptides per fraction (Figure 1b) helps evaluate the fractionation quality, and is in agreement with reported data [4,6]. A plot of the general distributions of these spans assists with further evaluation of IEF performance (Figure 1c). Every peptide, either unique or *n*-spanned, is given an arbitrary number as an identifier based on the width of its span. Peptides identified in each fraction (x-axis) are then sorted by their identifier and categorised by their *n*-span (unique, 2-span, 3-span, etc.; y-axis). The plot visualises the overall fractionation pattern of this IEF experiment and how different *n*-spanned categories contribute to overall peptide identifications in each fraction. In accordance with previous findings [3], the lowest separation performance was observed in the middle pH range (F6-F15) with Figure 1c showing contributions from unique up to 12 spanned peptides. Basic peptides (F19-F24) were found to have a slightly narrower span, whilst the highest focussing performance was observed in the acidic pH range (F1-F5) with most peptides not spanning more than 2 fractions. Of all peptide identifications, 72.1% were unique. The widest span corresponded to a 12-spanned peptide (sequence VADIGLAAWGR) consisting of a 10-span across the neutral pH range and a 2-span in the final basic fractions, which originated from the abundant protein S-adenosylhomocysteine hydrolase.

A total of 27.9% of all peptide identifications were found to be non-unique (Figure 1c), with peptide spans ranging from 2 to 12, which can be identified across adjacent fractions or contain gaps. In order to visualise the extent of this spread across all adjacent fractions only (89.4% of all the spreads in our data set), a Venn-like summary diagram was designed (Figure 1d). The areas of the circles are proportional to the number of peptides identified in a fraction and the areas of their intersection to the number of peptides in common (numbers are displayed were circles intersect). This pairwise comparison reinforces that in the acidic pH region, where circles intersect less, the peptide focussing performance is superior. For example, F3 and F4 had 37 peptides in common out of 322 and 265, respectively. In contrast, F10 and F11 in the neutral pH range had 146 peptides in common out of 298 and 290.

Another data display format was developed to help elucidate the nature of the spread between adjacent fractions (Figure 1e). Within each fraction, peptides are first sorted according to their estimated pI, then plotted as short horizontal segments whose length is proportional to their pI and assembled to columns which progressively become wider from top to bottom and left to right. Peptides shared across adjacent fractions are connected by a line. If fractions F1 and F2 were identical, their bar length would be equal and the lines in between all horizontal. Because of the progressive increase of the theoretical pI ranges from left to right, lines should ideally join peptides in common from the higher pI range at the bottom of one fraction to the lower pI range at the top of the next fraction, inducing only positive slopes. This pattern -where observed- is expected for a continuous separation method where discrete fractions were collected, which arbitrarily section individual peptide separation profiles. However, slopes of connecting lines may be negative and a general trend may not be discernable, when either the overlap is too large or discrepancies exist between estimated and experimental pIs.

Many peptides especially with neutral pI values have flat charge versus pH titration curves around their pI and hence are less likely to fractionate well in isoelectric focussing [18]. This tool calculates and extracts for each peptide a parameter that describes the length of the pH range where the net charge of the peptide is below a threshold value (*i.e*. ± 0.01). The connecting lines between the columns in Figure 1e, reflecting the peptide spanning between OF-FGEL fractions, are graded on a grey scale corresponding to the length of pH range where the net charge falls within the selected threshold margins (the darker the line, the wider the pH range). Figure 1e shows that this parameter, an intrinsic peptide property, is a major contributor to the poor experimental fractionation performance around the neutral pH range resulting in insufficient focussing. This facilitates a meaningful assessment of the fit between the theoretically expected and the experimentally observed peptide distributions across the OFFGEL fractions.

## Conclusions

In conclusion, a visualisation tool for peptide fractionation has been developed to support the evaluation of IEF data quality and may be implemented in proteomics research or device optimisation. Peptide *n*-spans across fractions can be determined, pairwise comparison between adjacent fractions quantified and the nature of spread elucidated. This tool is portable to other platforms (e.g. Bioconductor [19]) and transferable to other proteomics fractionation techniques such as ion exchange chromatography or other types of IEF.

## Availability and requirements

• **Project name: **iefviz

• **Project home page: **http://sourceforge.net/projects/iefviz/

• **Operating system: **Linux

• **Programming language: **Perl 5.8

• **Other requirement: **Gnuplot 4.2

• **License: **GNU GPL

## Authors' contributions

DOA implemented the tool. HN participated in the design and coordination of the work. MFO carried out the IEF and LC-MS/MS experiments. All authors drafted, read and approved the final manuscript.

## References

[B1] de Godoy LMF Olsen JV Cox J Nielsen ML Hubner NC Fröhlich F Walther TC Mann M Comprehensive mass-spectrometry-based proteome quantification of haploid versus diploid yeast Nature 2008 455 1251 1254 10.1038/nature07341 18820680

[B2] Michel PE Reymond F Arnaud IL Josserand J Giraud HH Rossier JS Protein fractionation in a multi-compartemental device using Off-Gel isoelectric focusing Electrophoresis 2003 24 3 11 10.1002/elps.200390030 12652567

[B3] Hubner NC Ren S Mann M Peptide separation with immobilized pI strips is an attractive alternative to in-gel protein digestion for proteome analysis Proteomics 2008 8 4862 4872 10.1002/pmic.200800351 19003865

[B4] Hörth P Miller CA Preckel T Wenz C Efficient Fractionation and Improved Protein Identification by Peptide OFFGEL Electrophoresis Molecular and Cellular Proteomics 2006 5 1968 1974 10.1074/mcp.T600037-MCP200 16849286

[B5] Fraterman S Zeiger U Khurana TS Rubinstein NA Wilm M Combination of peptide OFFGEL fractionation and label-free quantitation facilitated proteomics profiling of extraocular muscle Proteomics 2007 7 3404 3416 10.1002/pmic.200700382 17708596

[B6] Chenau J Michelland S Sibide J Seve M Peptides OF-FGEL electrophoresis: a suitable pre-analystical step for complex eukaryotic samples fractionation compatible with quantitative iTRAQ labeling Proteome Science 2008 6 9 10.1186/1477-5956-6-9 18302743 PMC2277393

[B7] Ernoult E Gamelin E Guette C Improved proteome coverage by using iTRAQ labelling and peptide OFFGEL fractionation Proteome Science 2008 6 27 10.1186/1477-5956-6-27 18851748 PMC2572582

[B8] Kyte J Doolittle RF A Simple Method for Displaying the Hydrophatic Character of a Protein Journal of Molecular Biology 1982 157 105 132 10.1016/0022-2836(82)90515-0 7108955

[B9] ExPASy http://www.expasy.ch/tools/pi_tool.html

[B10] Innovagen http://www.innovagen.se/custom-peptide-synthesis/peptide-property-calculator/peptide-property-calculator.asp

[B11] Perl http://www.perl.com

[B12] HTTP::Request http://github.com/gisle/libwww-perl

[B13] Gnuplot http://www.gnuplot.info

[B14] Tukey JW Exploratory Data Analysis 1977 Addison-Wesley

[B15] Area of Intersection of Two Circles http://mathforum.org/library/drmath/view/54785.html

[B16] Nelson DL Cox MM Lehninger Principles of Biochemistry 2004 W.H. Freeman

[B17] Heller M Ye M Michel PE Morier P Stalder D Jünger MA Aebersold R Reymond F Rossier JS Added Value for Tandem Mass Spectrometry Shotgun Proteomics Data Validation through Isolectric Focusing ofPeptides Journal of Proteome Research 2005 4 2273 2282 10.1021/pr050193v 16335976

[B18] Lam HT Josserand J Lion N Girault H Modeling the Isoelectric Focusing of Peptides in an OFFGEL Multicompartmental Cell Journal of Proteome Research 2007 6 1666 1676 10.1021/pr0606023 17397209

[B19] Bioconductor http://www.bioconductor.org

